# Supplementary material for: Influenza Virus Infection Model With Density Dependence Supports Biphasic Viral Decay
Source: Front Microbiol. 2018 Jul 10;9:1554. doi: 10.3389/fmicb.2018.01554 (PMC6048257; doi:10.3389/fmicb.2018.01554)
Supplement: Supplementary file 1 [file Presentation_1.PDF]

Supplementary Material: Influenza Virus Infection Model with Density Dependence Supports Biphasic Viral Decay

Amanda P. Smith, David J. Moquin, Veronika Bernhauerova, Amber M. Smith*

1 FITS OF THE STANDARD VIRAL KINETIC MODEL

The standard viral kinetic model is given by Equations (1)-(4) with $\delta(I_2) = \delta_s$ (subscript s denotes "standard") (Baccam et al., 2006). Figure S1 shows the model fits to viral load data from groups of mice infected with $75\ \mathrm{TCID}_{50}$ PR8 (see Main Text). The fits either included (dashed line) or excluded (solid line) the data at 8–9 d pi. In addition, we examined the goodness of fit when the initial condition for the infected cells ($I_1(0)$) or for virus ($V(0)$) was either fixed or estimated (see Table S1). The results illustrate that the standard viral kinetic model fails to fit the entire data set but can fit the data up to 7 d pi. In addition, fixing the initial number of infected cells $I_1(0)$ resulted in the best fit (Table S1).

There is a slight discrepancy in the estimate for δ_s compared to the linear regression estimate reported in the Main Text ($0.4\ \mathrm{d}^{-1}$). This is due to the inclusion of the data at 7 d pi in these fits. The slope of the data between 3–7 d pi is $-0.3\ \log_{10}\mathrm{TCID}_{50}/\mathrm{d}$ (compared to $-0.2\ \log_{10}\mathrm{TCID}_{50}/\mathrm{d}$ for 2–6 d pi), which correlates to $\delta_s = 0.7\ \mathrm{d}^{-1}$. Thus, the linear regression remains an approximation to the estimate of δ_s (Table S1).

Table S1. Parameters obtained from fitting the standard viral kinetic model (Equations (1)-(4) with $\delta(I_2) = \delta_s$) to viral titers from mice infected with $75\ \mathrm{TCID}_{50}$ PR8. The subset of data used in the fit is indicated. The initial number of infected cells ($I_1(0)$) or the initial amount of virus ($V(0)$) were fixed or estimated as indicated. For all estimations, the initial number of target cells ($T(0) = 10^7$ cells) and the initial number of productively infected cells ($I_2(0) = 0$ cells) were fixed. The sum of the squared residuals (SSR) and Akaike Information Criteria with small sample size correction ($\mathrm{AIC_c}$) are given. $\Delta\mathrm{AIC_c} < 2$ is considered statistically equivalent.

**Parameter**	**Value**				**Value**			
	Data: 4 h – 7 d				Data: 4 h – 9 d			
	$I_1(0)$		$V(0)$		$I_1(0)$		$V(0)$	
	fixed	estim.	fixed	estim.	fixed	estim.	fixed	estim.
β ($\times 10^{-5}$)	21.2	21.5	232.2	24.8	0.12	0.19	0.035	0.24
k	4.0	4.0	4.0	4.0	4.0	4.0	4.0	4.0
δ_s	0.7	0.7	0.4	0.7	3.2	2.9	3.1	2.8
p	0.25	0.25	151.2	0.24	9.75	13.0	188.8	10.9
c	1.0	1.0	1000	1.0	3.2	2.9	33.5	2.9
$I_1(0)$	75	72.1	0	0	75	7.5	0	0
$V(0)$	0	0	75	0.4	0	0	75	2.8
SSR	7.82	7.82	10.16	7.84	122.77	116.86	128.94	118.23
$\mathrm{AIC_c}$	39.82	95.82	41.91	95.84	50.08	64.58	50.57	64.70
$\Delta\mathrm{AIC_c}$		56.0	2.1	56.0		14.5	0.5	14.6

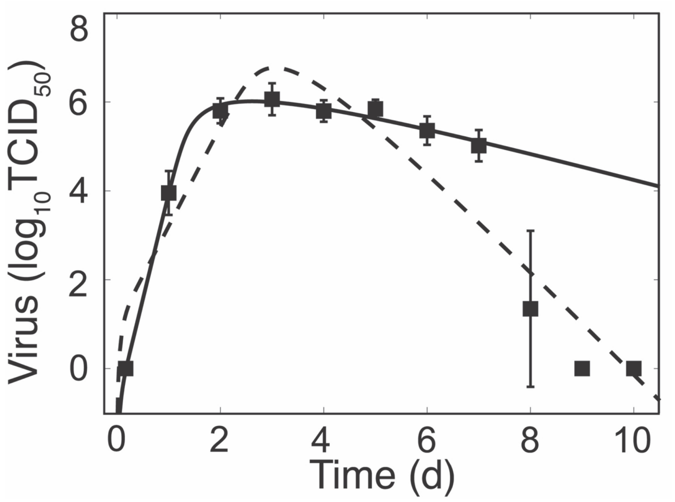

Figure S1: **Fit of the Standard Viral Kinetic Model**. Fit of the standard viral kinetic model (Equations (1)-(4) with $\delta(I_2) = \delta_s$) to viral titers from mice infected with 75 TCID$_{50}$ PR8. The initial number of infected cells was fixed at $I_1(0) = 75$ cells. Best-fit parameters are given in Table S1. Fits included (dashed line) or excluded (solid line) the data at 8-9 d pi.

2 PARAMETER ESTIMATION WITH ADAPTIVE SIMULATED ANNEALING

To estimate the parameters, we used the Adaptive Simulated Annealing (ASA) global search algorithm (Ingber, 1989, 1993; Černý, 1985). To increase accuracy of the solution, we first minimized the cost function $C(\theta)$ (see Main Text) using the ASA algorithm then used the *fmincon* subroutine in MATLAB to perform a local minimization. A brief explanation of the ASA algorithm and the pseudocode are included below.

The ASA evolves a single point $\boldsymbol{\theta} = (\theta_1, \ldots, \theta_N)$ in the N-dimensional parameter space. A random search is guided by the generating probability density function ($G(\theta_i^{\text{new}}, \theta_i^{\text{old}}, T_{i,\text{gen}}; 1 \leq i \leq N)$) that determines how a new state ($\boldsymbol{\theta}^{\text{new}}$) is created given the current state ($\boldsymbol{\theta}^{\text{old}}$) and the generating 'temperature' ($T_{i,\text{gen}}$) along each dimension of the state space (Θ). The generated point ($\boldsymbol{\theta}^{\text{new}}$) is accepted if it improves the value of the cost function, otherwise it is accepted with the probability $P(C(\boldsymbol{\theta}^{\text{old}}), C(\boldsymbol{\theta}^{\text{new}}), T_{\text{accept}})$. This probability is large at high temperatures T_{accept}, which enables the algorithm to escape from local minima. Sensitivities that differ along each dimension of the state space are possible and incorporated into the algorithm.

Pseudocode

1. An initial N-dimensional vector of parameters $\boldsymbol{\theta}$ is randomly generated. The initial temperature of the acceptance probability function ($T_{\text{accept}}(0)$) is set to $C(\boldsymbol{\theta})$. The initial temperatures of the parameter generating probability functions ($T_{i,\text{gen}}(0)$) are set to 1.0. The annealing times ($n_i \leq i \leq N$ and n_a) are set to 0.
2. A new point is generated as

$$\theta_i^{\text{new}} = \theta_i^{\text{old}} + g_i \left(U_i - L_i \right) \quad \text{for } 1 \leq i \leq N, \tag{S1}$$

where L_i and U_i are the lower and upper bounds of the parameter θ_i, respectively. g_i is calculated as

$$g_i = \mathrm{sgn}\left(u_i - \frac{1}{2}\right) T_{i,\mathrm{gen}}(n_i) \left(\left(1 + \frac{1}{T_{i,\mathrm{gen}}(n_i)}\right)^{|2u_i-1|} - 1\right) \tag{S2}$$

where u_i is a uniformly distributed random variable in $[0, 1]$. If any generated θ_i^{new} is outside of the bounds $[L_i,\ U_i]$, it is discarded and a new point is generated.

3. The value of the cost function $C(\boldsymbol{\theta}^{\mathrm{new}})$ is then evaluated. If $C(\boldsymbol{\theta}^{\mathrm{new}}) < C(\boldsymbol{\theta}^{\mathrm{old}})$, then $\boldsymbol{\theta}^{\mathrm{new}}$ is accepted. Otherwise, the acceptance probability function of $\boldsymbol{\theta}^{\mathrm{new}}$, given by

$$P_{\mathrm{accept}} = \frac{1}{1 + \exp\left(\frac{C(\boldsymbol{\theta}^{\mathrm{new}}) - C(\boldsymbol{\theta}^{\mathrm{old}})}{T_{\mathrm{accept}}(n_a)}\right)}, \tag{S3}$$

is calculated and a uniform random variable q is generated in $[0, 1]$. If $q < P_{\mathrm{accept}}$, then $\boldsymbol{\theta}^{\mathrm{new}}$ is accepted. Otherwise, it is rejected.

4. After every 500 generated points, reannealing takes place by calculating normalized sensitivities

$$s_i = \left|\frac{C(\boldsymbol{\theta}^{\mathrm{pert}}) - C(\boldsymbol{\theta}^{\mathrm{best}})}{\boldsymbol{\theta}^{\mathrm{pert}} - \boldsymbol{\theta}^{\mathrm{best}}}\right| \frac{\boldsymbol{\theta}^{\mathrm{best}}}{C(\boldsymbol{\theta}^{\mathrm{best}})}, \quad 1 \le i \le N \tag{S4}$$

The values indicate the relative importance of parameter set ($\boldsymbol{\theta}^{\mathrm{pert}} = \varepsilon\boldsymbol{\theta}^{\mathrm{best}}$, $\varepsilon > 0$) on the cost function ($C(\boldsymbol{\theta}^{\mathrm{pert}})$). Let $s_{\max} = \max\{s_i, 1 \le i \le N\}$. Each parameter generating temperature $T_{i,\mathrm{gen}}$ is scaled by a factor $s_{\max}/s_i$ and the annealing time n_i is reset according to

$$T_{i,\mathrm{gen}}(n_i) = \frac{s_{\max}}{s_i} T_{i,\mathrm{gen}}(n_i) \tag{S5}$$

$$n_i = \left(-\frac{1}{m} \log\left(\frac{T_{i,\mathrm{gen}}(n_i)}{T_{i,\mathrm{gen}}(0)}\right)\right)^N \tag{S6}$$

Because the choice of m in in the range $1 - 10$ is often adequate and has been shown to not critically influence performance of the ASA (Chen and Luk, 1999), we set m to 5. In addition, $T_{\mathrm{accept}}(0)$ is reset to the value of the last accepted cost function, $T_{\mathrm{accept}}(n_a)$ is reset to $C(\boldsymbol{\theta}^{\mathrm{best}})$, and the annealing time n_a is rescaled according to

$$n_a = \left(-\frac{1}{m} \log\left(\frac{T_{\mathrm{accept}}(n_a)}{T_{\mathrm{accept}}(0)}\right)\right)^N \tag{S7}$$

5. After every N_{gener} generated points, annealing takes place with

$$n_i = n_i + 1, \tag{S8}$$

$$T_{i,\mathrm{gen}}(n_i) = T_{i,\mathrm{gen}}(0) \exp(-m\, n_i^{1/N}), \quad 1 \le i \le N \tag{S9}$$

and

$$n_a = n_a + 1, \tag{S10}$$

$$T_{\text{accept}}(n_a) = T_{\text{accept}}(0)\exp(-m\, n_a^{1/N}); \tag{S11}$$

otherwise, repeat step (ii).

6. The algorithm is terminated if the value of the cost function remained within 0.1 for 5 successive reannealing steps or a preset maximum number of cost function evaluations has been reached. Otherwise, step (ii) is repeated. An example of one run of the ASA algorithm on Equations (1)-(5) (Main Text) is shown in Figure S2.

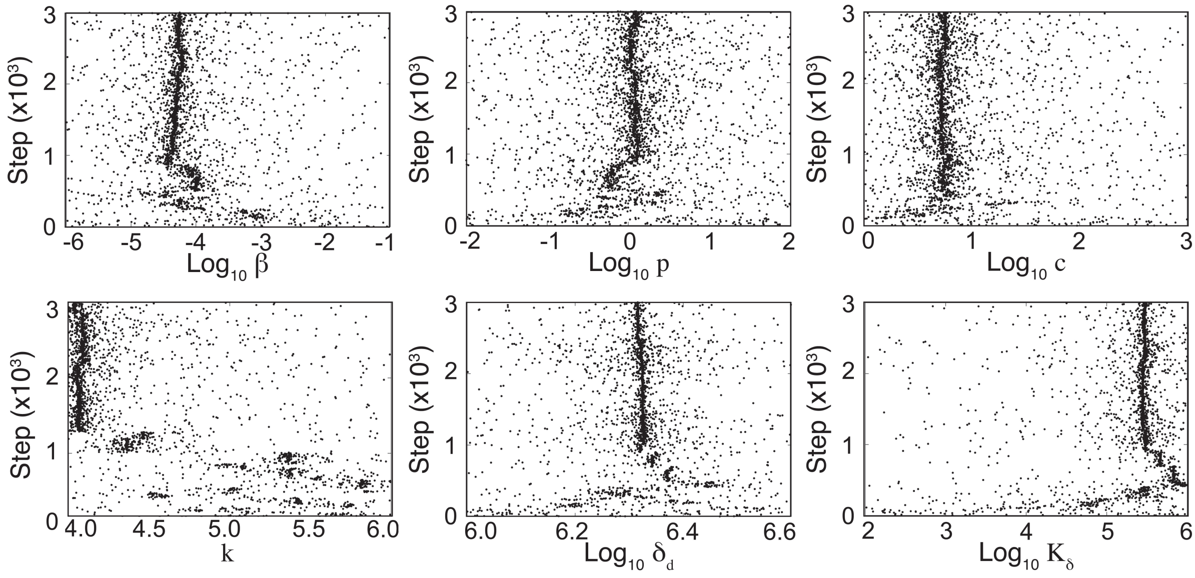

Figure S2: **ASA Parameter Search Pattern.** Parameter search pattern of the ASA algorithm while fitting the density-dependent viral kinetic model (Equations (1)-(5), Main Text) to viral titers from groups of mice infected with 75 TCID_{50} PR8. The algorithm initially accepts poor solutions with probability P, but the search becomes more directed over time. Due to the adaptive nature of the algorithm, parameters that are not well-defined will have less directed search patterns (e.g., k). Dots across the entire bounded region indicate a global search.

3 PARAMETER ENSEMBLES AND HISTOGRAMS

Figure S3 shows the parameter ensembles for the infected cell clearance parameter (δ_d), the half-saturation constant (K_δ), and the basic reproduction number (R_0). All other parameter ensembles and histograms are shown in Figure 2 in the Main Text.

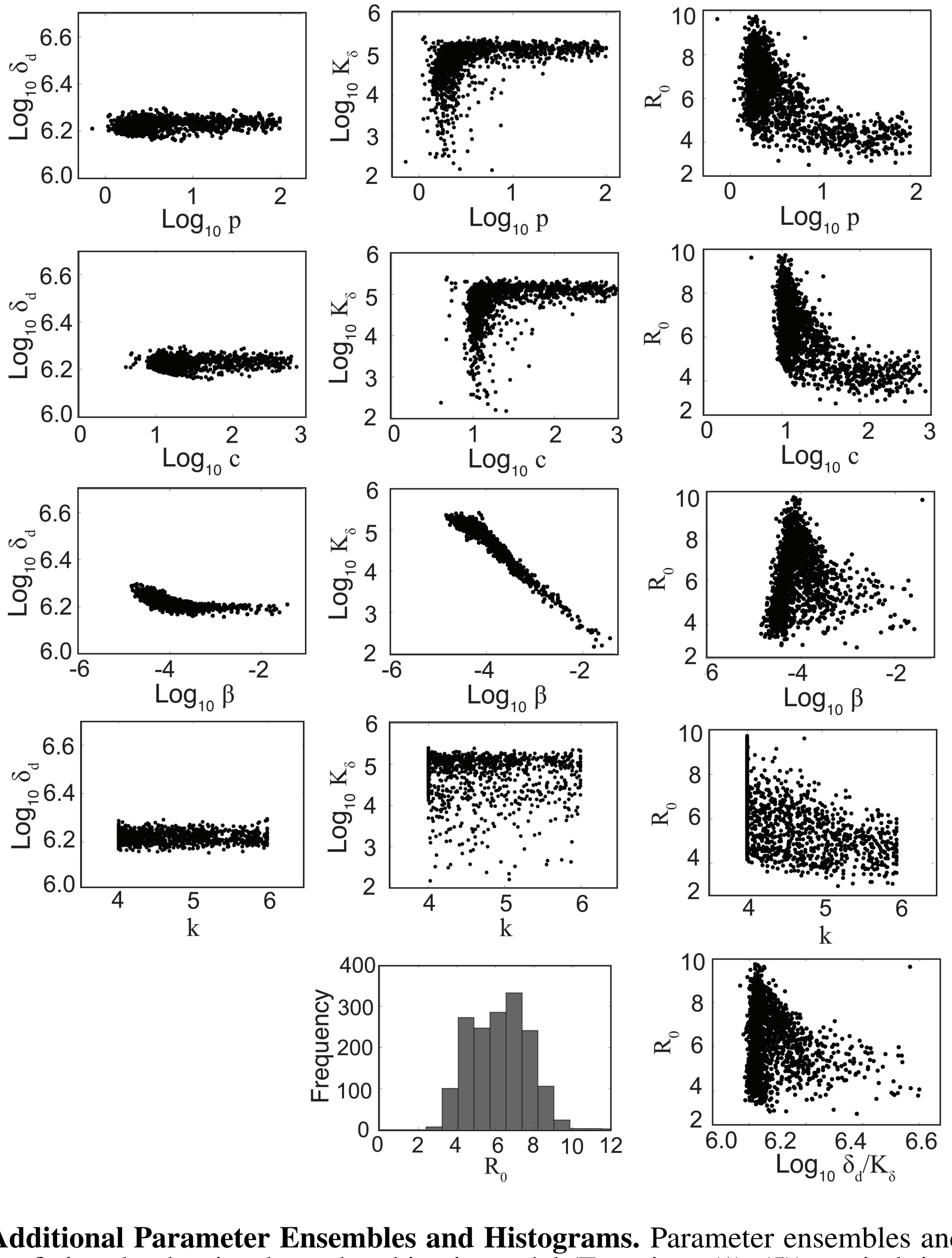

Figure S3: **Additional Parameter Ensembles and Histograms.** Parameter ensembles and histograms resulting from fitting the density-dependent kinetic model (Equations (1)–(5)) to viral titers from mice infected with 75 $TCID_{50}$ PR8. Ensemble plots for δ_d, K_δ, and R_0 are shown here. Ensembles for the other parameters, including the rate of infected cell clearance (δ_d/K_δ), are shown in Figure 2 in the Main Text.

REFERENCES

Baccam, P., Beauchemin, C., Macken, C., Hayden, F., and Perelson, A. (2006). Kinetics of influenza A virus infection in humans. *J. Virol.* 80, 7590–7599

Černý, V. (1985). Thermodynamical approach to the traveling salesman problem: An efficient simulation algorithm. *J. Optimiz. Theory App.* 45, 41–51

Chen, S. and Luk, B. (1999). Adaptive simulated annealing for optimization in signal processing applications. *Signal Process.* 79, 117–128

Ingber, L. (1989). Very fast simulated re-annealing. *Math. Comput. Model.* 12, 967–973

Ingber, L. (1993). Simulated annealing: Practice versus theory. *Math. Comput. Model.* 18, 29–57
